# Increasing compliance with low tidal volume ventilation in the ICU with two nudge-based interventions: evaluation through intervention time-series analyses

**DOI:** 10.1136/bmjopen-2015-010129

**Published:** 2016-05-26

**Authors:** Christopher P Bourdeaux, Matthew JC Thomas, Timothy H Gould, Gaurav Malhotra, Andreas Jarvstad, Timothy Jones, Iain D Gilchrist

**Affiliations:** 1 Intensive Care Unit, University Hospitals Bristol, Bristol, UK; 2 School of Experimental Psychology, University of Bristol, Bristol, UK; 3 NIHR CLARHC West, Bristol, UK

**Keywords:** decision-making, behavioural science, low tidal volume ventilation, nudge

## Abstract

**Objectives:**

Low tidal volume (TVe) ventilation improves outcomes for ventilated patients, and the majority of clinicians state they implement it. Unfortunately, most patients never receive low TVes. ‘Nudges’ influence decision-making with subtle cognitive mechanisms and are effective in many contexts. There have been few studies examining their impact on clinical decision-making. We investigated the impact of 2 interventions designed using principles from behavioural science on the deployment of low TVe ventilation in the intensive care unit (ICU).

**Setting:**

University Hospitals Bristol, a tertiary, mixed medical and surgical ICU with 20 beds, admitting over 1300 patients per year.

**Participants:**

Data were collected from 2144 consecutive patients receiving controlled mechanical ventilation for more than 1 hour between October 2010 and September 2014. Patients on controlled mechanical ventilation for more than 20 hours were included in the final analysis.

**Interventions:**

(1) Default ventilator settings were adjusted to comply with low TVe targets from the initiation of ventilation unless actively changed by a clinician. (2) A large dashboard was deployed displaying TVes in the format mL/kg ideal body weight (IBW) with alerts when TVes were excessive.

**Primary outcome measure:**

TVe in mL/kg IBW.

**Findings:**

TVe was significantly lower in the defaults group. In the dashboard intervention, TVe fell more quickly and by a greater amount after a TVe of 8 mL/kg IBW was breached when compared with controls. This effect improved in each subsequent year for 3 years.

**Conclusions:**

This study has demonstrated that adjustment of default ventilator settings and a dashboard with alerts for excessive TVe can significantly influence clinical decision-making. This offers a promising strategy to improve compliance with low TVe ventilation, and suggests that using insights from behavioural science has potential to improve the translation of evidence into practice.

**Strengths and limitations of this study**

- There are few studies that examine impact of behavioural insights (or ‘nudges’) on clinical decision-making and, to our knowledge, this is the first study to investigate the impact of default settings on ventilation practice.
- We examined the effect of the interventions on a large data set using hourly tidal volumes, a far greater frequency of sampling than achieved in most ventilation studies.
- The allocation of default ventilators was pragmatically randomised according to the availability of each ventilator.
- The dashboard evaluation was undertaken as a before-and-after comparison, and may be influenced by unmeasured factors.
- The study was undertaken in a single intensive care unit and, therefore, should be repeated in a large multicentre, randomised, controlled trial.

## Introduction

The translation of evidence-based interventions into clinical practice is inconsistent.^1^ The resulting variation in care can worsen outcomes.^2^ It is often assumed that simply presenting clinicians with information on best evidence will lead to adoption into practice and improvements in care. However, it is unlikely that this ‘rational’ model, which assumes that clinicians can integrate and hold in mind all the necessary information, is valid. Instead, decision-making is likely constrained by a range of cognitive, social and emotional factors.^3^

In the current paper, we investigate this problem with respect to low tidal volume (TVe) ventilation in the intensive care unit (ICU). Clinicians working in the ICU must make large numbers of time-constrained decisions in order to deliver optimal care to individual patients. It has been estimated that to comply with best available evidence for a complex patient on the ICU, intensivists must deliver 80–200 interventions daily,^4^ one of which is maintaining low TVes for patients on mechanical ventilation.

Randomised controlled trials and meta-analyses have shown that low TVes (ie, TVes<6 mL/kg ideal body weight (IBW), combined with appropriate positive end expiratory pressure (PEEP) adjustment, benefit ventilated patients,^5–8^ but the adoption of this strategy has been inconsistent.^9^ ^10^ A recent study found that only 37% of eligible patients received low TVes.^11^

Importantly, although physicians state they intend to use low TVes and implement them frequently or always, they often fail to deliver them in practice.^12^ ^13^

In order to implement appropriate TVes, clinicians must calculate IBW using the patient's height. They must then divide the volume of each breath by the IBW to give the format mL/kg IBW. Given the conditions clinicians operate under, it seems implausible that they have the cognitive capacity to constantly integrate this information and apply it to their patients as the rational model of decision-making suggests.^14^ In a previous study, we demonstrated that a large dashboard displaying TVes in real time, in a format that reduces cognitive effort, reduced mean TVe.^15^ In this study, we examine the response of clinicians to individual high TVe alerts over a 3-year period. We also examine the effect of setting an initial default on the ventilators that is consistent with low TVe targets.

## Methods

This prospective observational study was undertaken in the ICU at University Hospitals Bristol, UK, a closed-format tertiary medical and surgical ICU. The unit has used the Innovian Solution Suite clinical information system (CIS) (Draeger, Germany) since 2008. The CIS automatically collects all information relating to patient care including data from ventilators. This information is displayed on a computerised chart, and is also stored on a database (Microsoft SQL server, 2008). Doctors, some nurses and some physiotherapists can adjust the ventilators. The interventions targeted the entire clinical team. An entry on the staff communication book accompanied the introduction of the interventions. No additional staff training or education regarding low TVe ventilation was undertaken. The ICU does not employ respiratory therapists. The institutional review board waived the requirement for individual consent.

### Patients

Consecutive patients undergoing controlled mechanical ventilation were included in the study.

### Data collection and preprocessing

We collected anonymised data from 2144 ventilated patients between October 2010 and September 2014. Ventilation data for these patients were automatically recorded at least hourly in the CIS database. We preprocessed these data to avoid issues introduced by missing and irregularly sampled data. Irregularities in TVe time series data can arise from system errors, but also from changes in the clinical treatment of the patient. For example, a patient may be taken off the ventilator for a short time, or clinicians may request an increased sampling rate. Additionally, many patients will only be ventilated for a very short time.

If there were gaps in the time series that were >2 hours, we excluded these patients from further analysis. For gaps in the time series that were 2 hours or less, the time series were linearly interpolated. Sample rates higher than 1 hour were down-sampled to the standard 1 hour rate. Furthermore, to avoid biasing effects of ventilation duration on TVe trends, we ensured that all time series had the same length. We used a 20 hours continuous ventilation criterion, which is long enough to detect longer-term trends, while also including a substantial number of patients (944, which is approximately 44% of the total sample). In the default analysis, we used 20 hours from the initiation of ventilation. In the dashboard analysis, we used 20 hours from the first time a TVe breached 8 mL/kg IBW. Further analyses (not reported here) show that the results reported below are robust to changes in these preprocessing criteria.

TVe time series are asymmetrical about the mean and do not meet the assumptions of normality, partly because TVes are more strictly bounded at lower than higher values. Therefore, we describe these time series using robust statistics, plotting 50% trimmed means and estimating the SE using bootstrapping. We inferred the features of these time series by fitting parametric functions to each time series using non-linear regression (MATLAB, nlinfit, bisquare robust weighting function), and compared the 95% CIs on the best-fitting parameters (β weights). Non-overlapping CIs imply significantly different parameters. The entry of height is mandatory in the CIS, and this was used to calculate TVe in mL/kg IBW in all analysis.

### Intervention

#### Defaults

To study the effect of Default settings, patients were assigned to one of two groups—an Adjusted defaults group comprising 125 patients and a Control group comprising 182 patients. Patients in the Adjusted defaults group were ventilated using a Draeger Evita V500 ventilator. This ventilator can calculate IBW automatically when the patient's height is entered. We configured this ventilator to deliver TVes of 6 mL/kg IBW at the start of ventilation. This meant that unless clinicians chose to manually override this setting, a patient would be ventilated with TVes compliant with best evidence. If the height was not entered, the ventilator delivered TVes of 450 mL that is, the IBW was assumed to be 75 kg. By contrast, the patients in the Control group were ventilated using ventilators that did not have the ability to automatically deliver TVes based on height or IBW at the initiation of ventilation (Draeger Evita XL). The default ventilator settings in the control group were left as the factory setting which delivered TVes of 520 mL. Clinicians were able to alter ventilator settings at any time in both groups. The ICU had six of each type of ventilator during the duration of the study. The ventilators were stored centrally after use, and patients were allocated by nursing staff to the next available ventilator. The two types of ventilators have similar functions, modes and screen settings although the V500 is the newer model.

#### Dashboards

**Figure 1:**
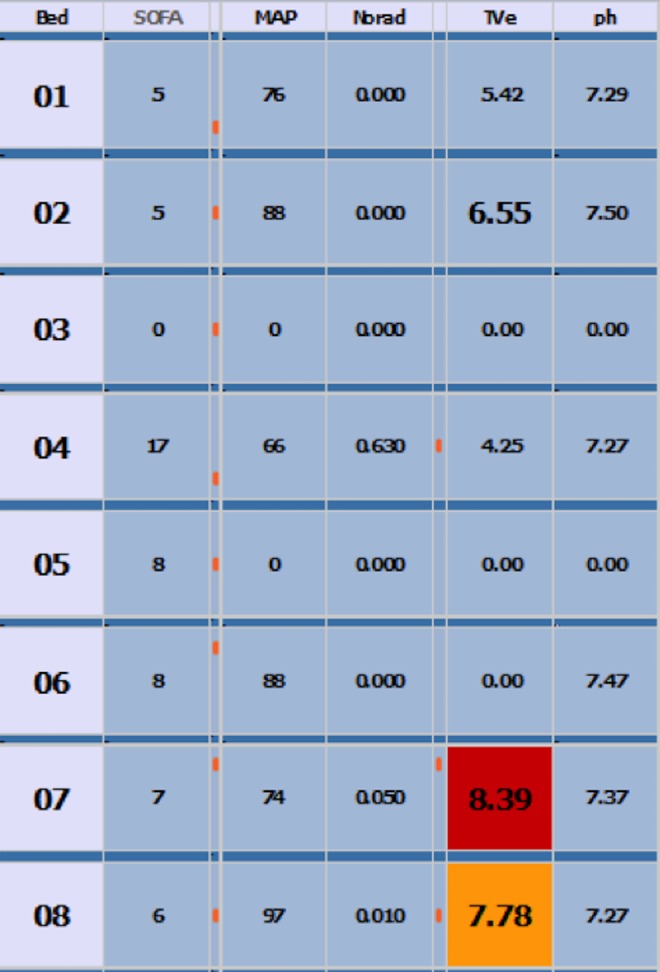
Dashboard appearance.

The ‘dashboard intervention’ provided salient visual cues to clinicians when TVes were high. Two large display screens were configured to display TVes derived from the CIS in the format mL/kg IBW (figure 1). Each screen displayed a matrix where the rows corresponded to patients and the columns corresponded to patient state variables, such as TVe. The screens were mounted on the wall at either end of the ICU and were easily visible. We focus on warnings produced when TVe >8, which resulted in the TVe cell for the patient in question turning red (the system also warned at TVe>6, resulting in a yellow warning) and analyse patients on controlled breathing modes only.

We analysed the effect of the red warning appearing on the dashboard by aligning (shifting) all time series to the first point at which TVe>8. The crossing of this threshold resulted in a warning on the dashboard display for the ‘post’ but not the ‘pre’ group. Thus, we compare time series for which there was a real warning when the threshold was crossed, to time series for which there was no warning, but for which there would have been one, had the dashboards been installed. We include only patients for which we have 20 hours of ventilator data after the threshold was first crossed (patients with <20 hours of ventilation data were excluded). This procedure resulted in a total of 553 TVe time series (76 pre and 477 post).

The dashboard intervention was introduced 1 year before the default intervention, and from that point, the interventions ran concurrently.

## Results

The baseline characteristics of patients included in the study are summarised in table 1.

**Table 1:** Study patient characteristics

Patient characteristic	
Age (mean (SD))	59.6 (±16.2)
Sex (%)	
Male	65
Female	35
Admission type (%)	
Emergency	82.4
Elective	17.6
APACHE II (mean (SD))	16.8 (±6.2)
ICU mortality (%)	26.7

APACHE, Acute Physiology And Chronic Health Evaluation; ICU, intensive care unit.

**Figure 2:**
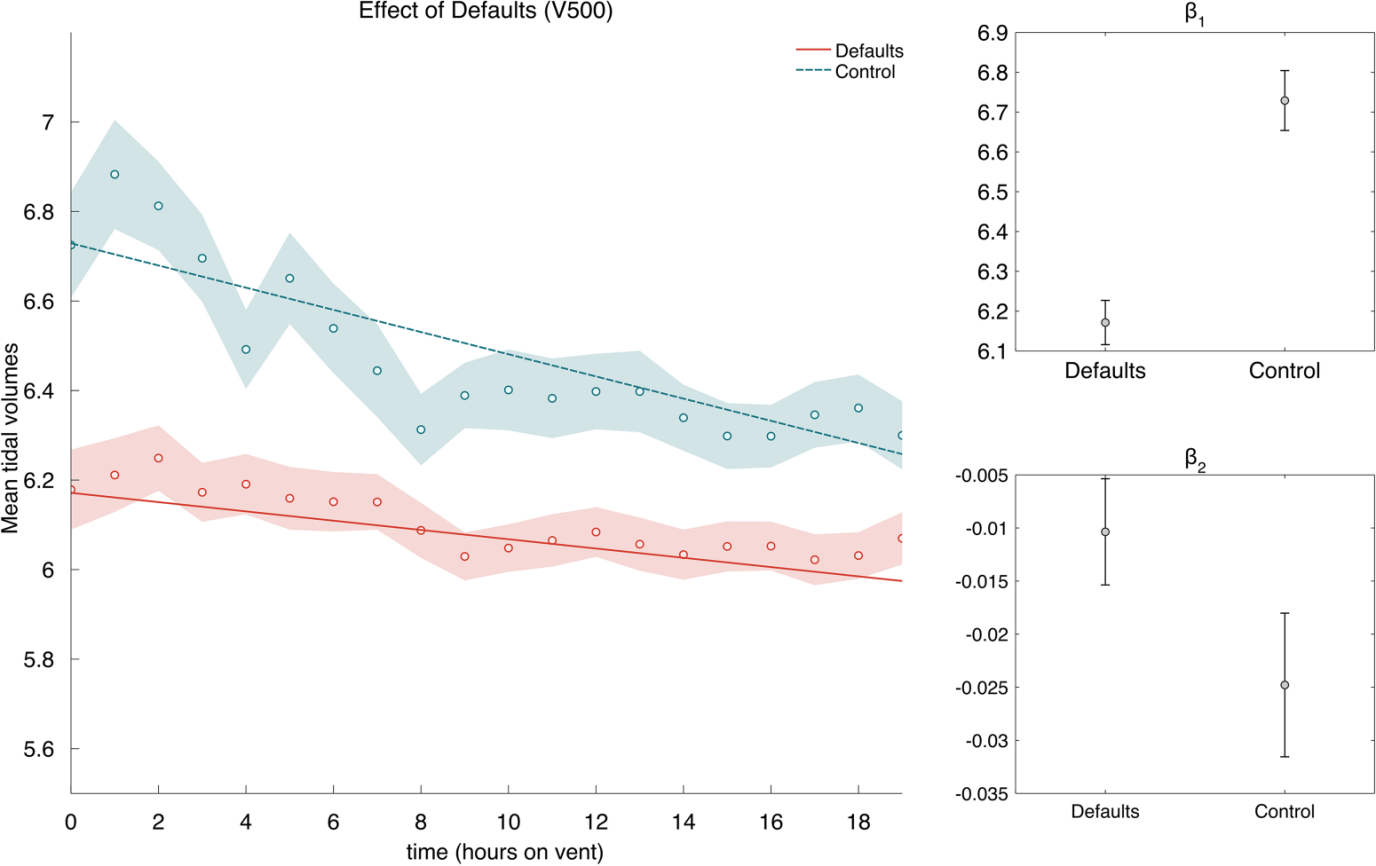
Effect of defaults and starting value on mean tidal volume. Tidal volume is displayed as a function of hours the patient was on ventilation. Averages are 50% trimmed means, and shaded regions are bootstrapped SEs. The lines are best-fit lines fit to the raw data. Intercepts 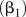 and slopes 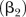 are shown for the best-fit lines in the main figure. Error bars are 95% CIs.

*Defaults*: Figure 2 compares the mean TVes for the Adjusted defaults group with those of the Control group. As can be seen, the average TVe was lower for the Adjusted defaults group (grand mean Defaults: 6.10, Control: 6.47).

We fitted the linear model, 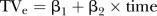, to the two time series, where the β weights 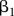 and 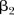 are the intercept and slope coefficients of the model. The best-fit lines are shown in figure 2A, and the best-fit coefficients are shown in figure 2B, C. The slope for both time series is negative, showing that TVe tends to decrease during the 20 hours after start of ventilation, and the slope for mean Control TVe is significantly larger than the mean Defaults TVe, showing a slower decrease for patients in the Adjusted defaults group. The fits from the linear model also suggest that the TVes in the control group remain significantly higher than in the Adjusted default group for the entire length of the analysis. We also observed that, when the initial TVe was low, there was a low deviation from the initial TVe for both Adjusted default and Control groups. However, when the initial TVe was high, there was a large deviation from the initial value for the Control group and a comparatively smaller deviation for the Adjusted default group.

**Figure 3:**
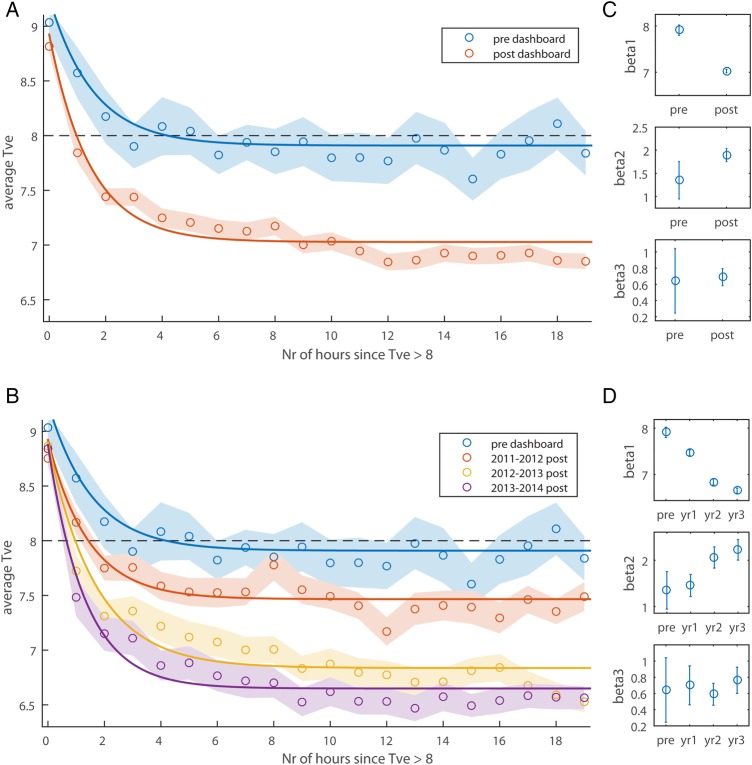
Tidal volume (TVe) following dashboard warning. Panels A and B show the average TVe as a function of the number of hours since TVe first crossed the warning threshold. A Shows the data split by predashboard and postdashboard, and B splits the dashboard data further into yearly postintervention periods. Averages are 50% trimmed means, and shaded regions are bootstrapped SEs. The lines are best-fit three-parameter exponential functions fit to the raw data. The black dashed line illustrates the threshold for dashboard warnings. C and D Show β weights for the best-fit lines in A and B respectively. Error bars are 95% CIs.

*Dashboard*: Figure 3A contrasts patients ventilated prior to the dashboard intervention with patients ventilated after the intervention (pre vs post). As can be seen, the main difference between the two TVe time series, is that the postgroup has lower TVe values following a threshold crossing (TVe >8) than the pregroup (ie, lower asymptotic TVe). Figure 3B shows an annual breakdown of the postdashboard data. As can be seen, TVe following a dashboard warning appears to decrease steadily over several years, with improvements as long as 3 years postintervention.

These trends were confirmed by exponential fits 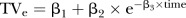, the best-fit β weights of which are shown in figure 3C. As can be seen, the CIs for 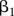 do not overlap between pre and post, nor do the CIs for 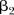. Thus, the two groups differ in both how quickly TVe drops 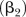, and how much it decreases 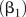, following dashboard warnings of high TVe values (TVe >8).

Each consecutive year resulted in lower TVe values than the previous year (see non-overlapping CIs 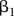, figure 3D). A significant increase in 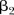 is also evident between year 1 and year 2 postdashboard, showing that the latter resulted in faster TVe drops following a warning. Using the model fits, we estimated the time it takes the TVe values to drop below threshold again, and this time decreases for each consecutive year: predashboard =4.2 hours; year 1=1.4 hours; year 2=0.95 hours and year 3=0.66 hours.

## Discussion

We assessed the association between TVe and two interventions designed to improve adherence to evidence-based ventilation targets, finding (1) that nudging clinicians by using best-practise default-settings resulted in improved best-practise compliance and (2) that alerting clinicians to excessive TVes was associated with a marked reduction in TVes.

Defaults are cognitively efficient, because the clinician need only make complex choices in cases that warrant a deviation from best practice. We know that default options have a powerful influence on behaviour in non-clinical settings including savings for retirement,^16^ and organ donation.^17^ Perhaps the best understood use of defaults in healthcare are those within electronic prescribing systems. The careful design of default options has been shown to dramatically change prescribing behaviour and improve compliance with evidence-based interventions.^18–20^ Although many default settings exist within the ICU, their role is poorly understood, and unless careful attention is applied to them they can result in harm,^21^ The default settings on ventilators lead patients to be ventilated on lower TVes for a persistent period of time. Patients who were on default ventilators had significantly lower TVes than patients who were not. Importantly, this pattern was evident from the initiation of ventilation. While the adjustment of default ventilator settings to reduce TVes has been recommended previously,^22^ to our knowledge, this study is the first to demonstrate a change in clinical practice as a result.

CISs offer an opportunity to reduce the cognitive effort required to comply with evidence and guidelines. While there is evidence for the use of computer-generated reminder systems in healthcare,^23–26^ there is less clarity about the effect on patient outcomes.^26–28^ As interventions, settings and methodologies are heterogeneous, it is difficult to conclude exactly which aspects of the systems are effective and in what settings. We designed our dashboards using insights from behavioural science and found that efficient display of routinely collected data had a significant impact on clinical practice. Excessive TVes triggered a visual alert in real time which, when compared with a control group with no alert, resulted in a more rapid fall in subsequent TVes and a lower asymptotic TVe. Interestingly, the data showed year-on-year improvements in TVe, with year 3 of the intervention showing markedly lower values than year 1.

A limitation of this study is that it involved only one ICU, and the evaluation of the dashboard intervention had a before-and-after design rather than a full randomised, control trial. TVes are slowly falling in practise, and the findings of our analysis of the dashboard intervention may be a reflection of this. However, note that in the default intervention, patients were randomly allocated to ventilators depending on the availability of different types of ventilator, and the analysis was not undertaken in a before-and-after fashion.

## Conclusions

This study has demonstrated that the application of simple interventions derived from behavioural science can significantly influence clinical decision-making regarding low TVe ventilation. The effect is stable with time, and in the case of dashboards, seems to improve year on year. Harnessing behavioural insights offers a promising strategy to improve the translation of evidence into clinical practice and deserves further study in randomised trials.
